# Bone health in neuroendocrine tumors and prognostic implications beyond skeletal metastases

**DOI:** 10.3389/fendo.2026.1882735

**Published:** 2026-07-01

**Authors:** Andrea Casabella, Iderina Hasballa, Anna Arecco, Mara Boschetti, Leonardo Della Sala, Davide Demontis, Alberto Sulli, Lara Vera, Alessandro Veresani, Diego Ferone, Sabrina Paolino, Manuela Albertelli

**Affiliations:** 1 Laboratory of Experimental Rheumatology and Academic Division of Clinical Rheumatology, Universita degli Studi di Genova Dipartimento di Medicina Interna e Specialita Mediche, Genoa, Italy; 2 Rheumatology Unit, IRCCS AOM Ospedale Policlinico San Martino, Genoa, Italy; 3 Endocrinology Unit, Università degli Studi di Genova, Dipartimento di Medicina Interna e Specialità Mediche, Genoa, Italy; 4 Endocrinology Unit, IRCCS AOM Ospedale Policlinico San Martino, Genoa, Italy

**Keywords:** neuroendocrine neoplasms, osteopenia/osteoporosis, progression-free survival, sarcopenia, TBS, vitamin D

## Abstract

**Background:**

The potential impact of NET (neuroendocrine tumor)-related disease burden on musculoskeletal health remains inconclusive.

**Aim:**

To assess the musculoskeletal status, its prognostic relevance and association with tumor aggressiveness in NET patients.

**Materials and methods:**

This cross-sectional study included 41 patients with grade (G) 1, 2, or 3 gastroenteropancreatic (GEP) and lung NETs. Among them, 38 were selected for comparison with 47 healthy controls matched for age, sex and body mass index (BMI). The musculoskeletal health was assessed by dual-energy X-ray absorptiometry (DXA) scan.

**Results:**

Within the NET group (median age=72 years old, 46% women), degraded TBS (trabecular bone score) was found in 71% of patients, with osteopenia affecting up to 59% at the femoral neck. The prevalence of hypovitaminosis D was 68%, whereas of low RSMI (relative skeletal muscle index) suggestive of sarcopenia 37%. Patients with advanced-stage NETs showed significantly lower L1-L4 BMD (bone mineral density), L1-L4 T-score, L1-L4 Z-score, 25-hydroxyvitamin D [25(OH)D] levels and BMI than those with earlier-stage disease. G2 NETs were associated with worse L1-L4 BMD, L1-L4 T-score, total hip T-score and diaphysis BMD than G1 NETs. In multivariate analysis, higher 25(OH)D levels and BMI were independently associated with longer progression-free survival (PFS), whereas higher Ki-67 was associated with shorter PFS. Correlation analyses showed inverse associations between Ki-67 and total hip T-score (rho = -0.314, p=0.048) and diaphysis BMD (rho = -0.354, p=0.025); age at NET diagnosis correlated with poorer bone parameters, whereas higher BMI was associated with better bone indices and RSMI. Compared with healthy controls, NET patients had significantly lower TBS, regardless of BMD, T-score or Z-score.

**Conclusion:**

NET patients showed a substantial burden of musculoskeletal impairment, with trabecular microarchitecture deterioration detectable despite BMD versus healthy controls. Advanced disease stage, higher grade and systemic treatment were associated with poorer bone health. Increased vitamin D levels and BMI were independently associated with longer PFS, supporting a potential relationship between nutritional-metabolic status and oncological outcomes. Low muscle mass was also frequent, although not significantly associated with tumor aggressiveness. These exploratory findings highlight the need for structured musculoskeletal assessment in NET patients and require validation in larger prospective studies.

## Introduction

Neuroendocrine neoplasms (NENs) are a heterogeneous group of tumors arising from neuroendocrine cells, most commonly of gastroenteropancreatic (GEP) and lung origin, characterized by increasing incidence and prevalence over the past decades (1). NENs may vary significantly in histopathological and molecular profile, functional status, and clinical course. Based on the morphological differentiation, NENs are distinguished into well-differentiated neuroendocrine tumors (NETs) and poorly differentiated neuroendocrine carcinomas (NECs). While NECs are intrinsically considered high grade (G) (G3, Ki67 > 20%), NETs are further classified as G1, G2, or G3 based on the Ki-67 proliferative index and/or mitotic count. Lung NENs, instead, are categorized according to necrosis and mitotic activity into typical carcinoid (TC), atypical carcinoid (AC), small cell lung cancer (SCLC) and large cell neuroendocrine carcinoma (LCNEC); of these, TC and AC are regarded as NETs, whereas SCLC and LCNEC as NECs (2). Generally, the majority of NENs are non-functioning and occur sporadically. In about 5% of cases, however, NENs may be associated with hereditary syndromes such as multiple endocrine neoplasia 1 (MEN 1), multiple endocrine neoplasia 4 (MEN 4), Von Hippel Lindau (VHL) syndrome, tuberous sclerosis (TSC) or neurofibromatosis 1 (NF 1) (3). At present, surgery represents the only potentially curative treatment for well-differentiated and localized NENs. In advanced stages, when surgery is not feasible, available medical approaches include somatostatin analogues (SSAs), radioligand therapy (RLT), targeted agents and chemotherapy (4, 5).

The prevalence of synchronous distant metastasis at the time of NEN diagnosis has been reported to range from 20.8% to 43%, depending on the population-based registry, primary site, and histopathological spectrum of NENs (6, 7). In this context, although less frequent than liver and lymph node involvement, bone metastases (BM) represent a clinically meaningful component of disease burden and are regarded as a poor prognostic factor (8, 9). Emerging evidence, however, suggests that NEN patients may be susceptible to skeletal fragility beyond overt BM development, due to other mechanisms such as vitamin D deficiency, nutritional imbalance, hormonal syndrome, inflammation and NEN-related treatments. In this setting, chronic gastrointestinal symptoms, malabsorption, nutritional impairment and disease burden may also be plausible contributors to eventual musculoskeletal impairment (10).

However, the available evidence on both skeletal muscle and bone health in NENs, regardless of BM, remains limited, entirely retrospective and mainly focused on NENs of GEP origin. Despite increasing recognition of musculoskeletal impairment in NENs, standardized recommendations for the screening and management of musculoskeletal impairment in this population are still lacking. Our study was designed to explore potential associations between NET-related disease burden, systemic treatment exposure and musculoskeletal health in a cohort of patients affected by GEP and lung NETs.

## Materials and methods

### Study design and population

This retrospective, cross-sectional observational study was conducted through a collaboration between the Endocrinology and Rheumatology Units of IRCCS Policlinico San Martino of Genoa. Among the 78 NET patients referring to the Endocrinology Unit between 2000 and 2023 and meeting the inclusion criteria, 41 were included in the final analysis, as only these patients had complete medical records and recent dual-energy X-ray absorptiometry (DXA) assessment.

The inclusion criteria were: (I) age between 18 and 85 years old; (II) cytological or histopathological diagnosis of well-differentiated neuroendocrine neoplasm (NET), G1-2-3, of GEP or lung origin; (III) NET patients undergoing active surveillance, surgery and/or medical systemic treatment with SSAs and/or RLT.

The exclusion criteria were: (I) age < 18 years old or > 85 years old; (II) Patients with advanced and progressive NETs receiving target therapies (everolimus, sunitinib) and/or chemotherapy; (III) Patients with neuroendocrine carcinoma or other NENs such as pheochromocytoma or paraganglioma, medullary thyroid cancer, Merkel cell carcinoma; (IV) concurrent comorbidities impacting bone health (i.e. primary hyperparathyroidism [PHPT], Paget disease, adrenal insufficiency, hyperthyroidism); (V) confirmed diagnosis of MEN 1 or MEN 4 syndrome, due to the higher frequency of PHPT associated with these syndromes; (VI) ongoing treatments potentially affecting bone health, including anti-osteoporotic agents and prolonged glucocorticoid therapy (>6 months); (VII) incomplete medical records.

Additionally, a control group (CT) of 47 healthy individuals was identified according to the following inclusion criteria: (I) absence of concomitant diseases or chronic treatments potentially influencing bone health; (II) serum 25-hydroxyvitamin D [25(OH)D] levels ≥30 ng/mL, regardless of vitamin D supplementation, to define a metabolically healthy and vitamin D-replete reference population.

Among the 41 NET patients, a subgroup of 38 patients matched to healthy controls for age, sex, and BMI was selected for the comparative DXA analysis.

The study was approved by the local Ethics Committee and was conducted in accordance with the approved protocol, n. 377/2023 – DB id 13324.

Informed consent was obtained from all participants, and data were collected anonymously.

### Study outcomes

The primary outcomes of the study were (I) to explore bone health in patients with GEP and lung NETs through DXA-derived parameters, (II) to compare these measures with those observed in age-, sex- and BMI-matched healthy controls, and (III) to investigate potential correlations between bone status and tumor aggressiveness, assessed in terms of disease stage and grading, and NET-related medical therapy (SSA/RLT).

The secondary outcomes included the evaluation of (I) DXA-derived skeletal muscle mass using relative skeletal muscle index (RSMI); (II) vitamin D status and its potential impact on NET prognosis; (III) potential associations of DXA-derived parameters and nutritional-metabolic status, assessed in terms of BMI and glycometabolic profile, with progression-free survival (PFS).

PFS was defined as the time interval (months) from NET diagnosis to disease recurrence or progression according to RECIST (Response Evaluation Criteria in Solid Tumors) v1.1 criteria after first-line therapy (surgery or SSA), patients’ death for any cause, or last available follow-up in patients without disease progression.

### Study protocol

#### Bone health assessment

All participants underwent DXA analysis at the Osteoporosis Center of the Rheumatology Unit, IRCCS Ospedale Policlinico San Martino. Scans were performed using a Lunar Prodigy densitometer (GE Lunar, Madison, WI, USA) with enCORE software (GE Healthcare), version 18. Bone mineral density (BMD, g/cm²), T-score, and Z-score were assessed at the lumbar spine (L1-L4) and proximal femur, including the femoral neck (FN), Ward’s triangle, trochanter, and total hip. Trabecular bone score (TBS) and TBS-adjusted lumbar spine T-score were derived using TBS iNsight software (Medimaps Group/GE Healthcare, Needham, MA, USA; version 2.1.0.0). TBS values were interpreted according to previously proposed thresholds: normal (TBS≥1.350), partially degraded (1.200<TBS<1.350), and degraded (TBS ≤ 1.200) (11, 12).

#### DXA-derived body composition assessment

A subset of 27 NET patients also underwent whole-body DXA for body composition analysis at the time of bone evaluation, to assess total mass, lean mass, fat mass, bone mineral content (BMC), and BMD in specific body areas (head, upper limbs, lower limbs, trunk, spine, ribs, and pelvis). RSMI was calculated according to the Baumgartner equation as appendicular lean mass (upper and lower limbs, Kg) divided by height squared (m²). Based on EWGSOP (European Working Group on Sarcopenia in Older People) criteria, the cut-off values for low muscle mass potentially suggestive of sarcopenia were <7.0 Kg/m² for men and <5.5 Kg/m² for women (13).

#### Clinical, biochemical and histopathological data of NET patients

For each patient, clinico-histopathological data, including primary tumor site, grade, Ki-67 index, mitotic count, necrosis, tumor size, functional status, and disease stage, were collected from the time of NET diagnosis to DXA-assessment. Disease stage was categorized as stage I-II for localized NETs without nodal involvement, stage III for loco-regional nodal involvement, or stage IV for advanced NETs with distant metastasis. Disease recurrence or disease progression assessed according to RECIST v.1.1 criteria, as well as oncologic treatments (surgery, SSA, RLT) received up to DXA evaluation, were also recorded. To ensure temporal consistency with DXA findings, BMI and biochemical parameters—including calcium–phosphate metabolism, parathyroid hormone (PTH), 25(OH)D, renal function, and glycolipidic profile—were collected within 6 months of DXA assessment, thereby providing a cross-sectional, comprehensive overview of bone health and nutritional-metabolic status.

The sufficiency, insufficiency and deficiency of 25(OH)D levels were defined, respectively, for serum values ≥30 ng/mL, 20–29 ng/mL, and <20 ng/mL.

### Statistical analysis

All statistical analyses and graphical representations were performed using RStudio and IBM SPSS Statistics, version 22. Continuous variables were tested for normality using the Shapiro–Wilk test and are presented as mean, range and standard deviation (SD) or median and 95% confidence interval (CI). Categorical variables are expressed as absolute numbers and percentages. Between-group comparisons were performed using the Student’s t-test or Mann–Whitney U test for continuous variables, depending on data distribution, and the χ² test or Fisher’s exact test for categorical variables. For comparisons among more than two groups, one-way ANOVA or Kruskal–Wallis test was applied, followed by *post hoc* analysis when appropriate. Correlation analyses between continuous variables were assessed using Pearson’s or Spearman’s correlation coefficients, according to data distribution. Survival analysis was conducted using the Kaplan–Meier method, and differences between groups were evaluated using the log-rank test. Cox proportional hazards regression analysis was performed to identify potential predictors of survival outcomes. Variables with p <0.05 at univariate analysis were included in the multivariate model. A two-sided p-value <0.05 was considered statistically significant.

## Results

### Study population: NET patients

A total of 41 NET patients, including 19 (46%) women and 22 (54%) men, were enrolled. The median age was 64 years (95% CI: 58-66) at NET diagnosis and 72 years (95% CI: 69-74) at the time of enrolment. The median BMI was 25.1 Kg/m² (95% CI: 23.9–26.9), with values classified as normal weight in 44% (n=18) of patients, overweight in 39% (n=16), obesity in 15% (n=6) and underweight in 2% (n=1). Regarding metabolic comorbidities, impaired fasting glucose and diabetes were documented, respectively, in 16 (39%) and 10 (24%) individuals, while hypertriglyceridemia (≥ 150 mg/dL) was observed in 6 cases (15%). About 25(OH)D serum concentration, sufficient levels were found in 13 patients (32%), whereas insufficiency and deficiency were recorded in 18 (44%) and 10 (24%), respectively. At the time of DXA evaluation, vitamin D supplementation was being administered to the majority of the NET cohort (n=26; 63%). Furthermore, 8 subjects (20%) had a history of skeletal fractures; however, the available data did not allow for a definitive classification of these events as fragility-related.

As regards NET origin, 88% (n=36) of individuals were affected by GEP-NET, whereas 12% (n=5) by lung NET. Among GEP-NETs, the ileum was the most frequent primary site (n=15/36; 42%) followed by pancreas (n=14/36; 39%), duodenum (n=3/36; 8%), appendix (n=2/36; 5%), stomach (n=1/36; 3%), and rectum (n=1/36; 3%). About GEP-NET grading, the majority (58%, n=21) were G1, while 36% (n=13) G2 and 3% (n=1) G3; grading was unknown only in 1 (3%) case. Lung NETs, instead, were classified as typical in 4 (80%) cases and atypical in 1 (20%) case. The median Ki-67 index value was 2% (95% CI:1-3), whereas the median dimension of the primary NET was 16 mm (95% CI: 12-23). At NET diagnosis, 46% (n=19) of patients had stage I-II NET, 27% (n=11) stage III, whereas 27% (n=11) stage IV. Within the stage IV NET subgroup, liver metastases were observed in all patients, while bone metastases were identified in only 1 individual at baseline. During follow-up, BMs were subsequently observed in 5 additional patients. Moreover, among all NET subjects, 3 (7%) had functioning NETs, specifically carcinoid syndrome.

Regarding oncological therapy, most patients (n=36; 88%) underwent surgical resection of the primary NET, with complete remission achieved in 14 patients (34%) at the last follow-up (group 1). Disease recurrence following surgery, instead, was documented in 22 (54%) individuals, who required medical treatment with SSAs; of these, 7 also received third-line therapy with RLT due to further disease progression (group 2). Active surveillance was adopted in 5 (12%) patients with non-functioning pancreatic NETs smaller than 10 mm.

Patients’ demographic and anthropometric characteristics, as well as the clinicopathologic features of NETs, are presented in **Table 1**, while biochemical parameters are illustrated in **Table 2**.

**Table 1:** Patients’ characteristics and NET-related clinical-histopathological and therapeutic data.

Patients’ clinical and pathological characteristics			Overall NET cohort (n=41)
Patients’ anthropometric and demographic data	Sex (F; M)		F 19 (46%); M 22 (54%) M/F=1.2/1
	Median age (95% CI, yo)		72 (69–74)
	Median age at NET diagnosis (95% CI, yo)		64 (58-66)
	Median BMI (95% CI; Kg/m2)		25.1 (23.9-26.9)
Metabolic comorbidities	Diabetes mellitus (n; %)		10 (24%)
	Impaired fasting glucose (n; %)		16 (39%)
	Hypertriglyceridemia (n; %)		6 (15%)
NET origin (n; %)	GEP	Ileum	15 (42%)
		Pancreas	14 (39%)
		Duodenum	3 (8%)
		Appendix	2 (5%)
		Stomach	1 (3%)
		Rectum	1 (3%)
		Total	36 (88%)
	Lung		5 (12%)
GEP-NET grade (n; %)	G1		21 (58%)
	G2		13 (36%)
	G3		1 (3%)
	Unknown		1 (3%)
Lung carcinoids (n; %)	Typical		4 (80%)
	Atypical		1 (20%)
Functioning status of NETs (n; %)	Functioning (carcinoid syndrome)		3 (7%)
	Non-functioning		38 (93%)
Stage at diagnosis (n; %)	I-II		19 (46%)
	III		11 (27%)
	IV		11 (27%)
Bone metastasis	At diagnosis (n; %)		1 (2%)
	During follow-up (n; %)		5 (12%)
NET therapy (n; %)	Active surveillance		5 (12%)
	Surgery alone		14 (34%)
	Surgery followed by medical therapy		22 (54%)

NET, neuroendocrine tumor; F, female; M, male; yo: years old; BMI, body mass index; GEP, gastroenteropancreatic; G, grade; n: number; CI, confidence interval.

**Table 2:** Biochemical data of NET patients.

Serum biochemical variable		Median (95% CI)	
25(OH)D (ng/mL)	Sufficiency (n; %)	26.0 (23.9 – 29.0)	13 (32%)
	Insufficiency (n; %)		18 (44%)
	Deficiency (n; %)		10 (24%)
Calcium (mg/dL)		9.3 (9.2 - 9.4)	
Calcium corrected for albumin (mg/dL)		9.1 (9.0 - 9.2)	
Phosphorus (mg/dL)		3.0 (2.9 - 3.1)	
PTH (ng/L)		43 (39 - 53)	
Creatinine (mg/dL)		0.9 (0.8 – 1.0)	
eGFR (ml/min)		81 (76 - 88)	
Albumin (g/dL)		4.3 (4.2 - 4.4)	
Total cholesterol (mg/dL)		178 (165 - 198)	
HDL cholesterol (mg/dL)		58 (51 - 65)	
LDL cholesterol (mg/dL)		100 (87 - 110)	
Triglycerides (mg/dL)		108 (73 - 115)	
Glycemia (mg/dL)		98 (93 - 116)	
HbA1c (%)		6 (5.6 - 6.2)	

NET, neuroendocrine tumor; 25(OH)D, 25-hydroxyvitamin D; PTH, parathyroid hormone; eGFR, estimated glomerular filtration rate; HDL, high-density lipoprotein; LDL, low-density lipoprotein; HbA1c, glycated hemoglobin; CI, confidence interval.

About DXA examination, based on lumbar spine (L1-L4) T-score, osteopenia was documented in 8 (20%) individuals, whereas osteoporosis in 2 (5%). FN T-score was suggestive of osteopenia and osteoporosis in 24 (59%) and 3 (7%) cases, respectively. Notably, 71% (n=29) of NET patients showed L1-L4 TBS values suggestive of degraded trabecular bone structure, with 20 (69%) and 9 (31%) cases exhibiting respectively partially and fully degraded microarchitecture. Furthermore, among the 27 patients undergoing DXA-evaluation of body composition, 10 (37%; 7 men and 3 women) had RSMI values indicative of low muscle mass suggestive of sarcopenia. Nevertheless, the lack of data on muscle strength and physical performance precluded a definitive diagnosis of sarcopenia based on the EWGSOP criteria. NET female and male subgroups differed significantly for several bone parameters, RSMI, as well as for both total lean and fat mass, despite comparable age (respectively, median 71 years old, 95% CI: 59–74 vs 73 years old, 95% CI: 69-74; p=0.556) and BMI (respectively, median 24.7 Kg/m^2^, 95% CI: 21.2-27.7 vs 25.9 Kg/m^2^, 95% CI: 24.1-28.3; p=0.513). More detailed information regarding DXA evaluation is illustrated in **Table 3**.

**Table 3:** DXA-derived parameters in NET patients.

DXA-derived parameter	NET patients (n=41) (median; 95% CI)	NET females (n=19) (median; 95% CI)	NET males (n=22) (median; 95% CI)	P-value
L1-L4 BMD (g/cm2)	1.19 (1.13; 1.24)	1.12 (1.03; 1.20)	1.27 (1.19; 1.36)	0.001
L1-L4 BMC (g)	72.88 (61.78; 76.95)	55.67 (53.22; 62.14)	78.47 (74.41; 97.72)	< 0.001
L1-L4 T-score	0.00 (-0.52; 0.32)	-0.50 (-1.31; 0.20)	0.45 (-0.30; 1.20)	0.010
L1-L4 Z-score	0.90 (0.45; 1.62)	1.30 (0.06; 1.94)	0.75 (-0.10; 1.52)	0.814
L1-L4 TBS	1.29 (1.23; 1.33)	1.29 (1.23; 1.32)	1.31 (1.22; 1.36)	0.676
FN BMD (g/cm2)	0.85 (0.80; 0.89)	0.84 (0.77; 0.93)	0.85 (0.79; 0.91)	0.917
FN BMC (g)	4.72 (4.38; 5.09)	4.40 (3.94; 5.31)	4.86 (4.53; 5.10)	0.497
FN T-score	-1.40 (-1.90; -1.05)	-1.20 (-1.90; -0.73)	-1.65 (-2.10; -1.09)	0.360
FN Z-score	-0.10 (-0.50; 0.40)	0.40 (-0.10; 0.70)	-0.45 (-0.80; 0.20)	0.028
Total hip BMD (g/cm2)	0.94 (0.91; 1.02)	0.95 (0.87; 1.09)	0.94 (0.89; 1.03)	0.794
Total hip BMC (g)	32.84 (29.95; 34.61)	32.74 (25.78; 38.27)	33.39 (30.10; 35.68)	0.814
Total hip T-score	-0.70 (-1.20; -0.35)	-0.60 (-1.22; 0.00)	-0.75 (-1.20; -0.39)	0.638
Total hip Z-score	0.40 (0.18; 0.78)	0.40 (-0.10; 0.70)	0.35 (0.08; 0.67)	0.234
Trochanter BMD (g/cm2)	0.79 (0.73; 0.83)	0.79 (0.69; 0.93)	0.80 (0.73; 0.85)	0.948
Trochanter BMC (g)	10.92 (9.53; 12.46)	10.99 (8.46; 13.03)	10.68 (8.91; 12.53)	0.875
Trochanter T-score	-0.80 (-1.02; -0.28)	-0.60 (-0.02; 0.07)	-0.85 (-1.61; -0.59)	0.244
Trochanter Z-score	0.10 (-0.30; 0.32)	0.40 (-0.30; 0.95)	-0.05 (-0.70; 0.20)	0.043
Diaphysis BMD (g/cm2)	1.13 (1.09; 1.23)	1.13 (1.12; 1.32)	1.11 (1.06; 1.23)	0.278
Diaphysis BMC (g)	16.68 (15.56; 17.70)	16.37 (14.19; 19.77)	16.79 (16.13; 17.95)	0.875
Head BMD (g/cm2)	2.17 (1.92; 2.25)	2.08 (1.69; 2.32)	2.23 (1.95; 2.29)	0.395
Head BMC (g)	477.00 (402.19; 516.98)	463.00 (358.54; 537.19)	474.36 (431.34; 539.62)	0.472
Upper limbs BMD (g/cm2)	0.98 (0.87; 1.02)	0.82 (0.76-0.92)	1.03 (1.01; 1.04)	<0.001
Upper limbs BMC (g)	318.00 (248.43; 371.47)	223.00 (196.00; 261.00)	373.00 (330.71; 403.56)	<0.001
Lower limbs BMD (g/cm2)	1.17 (1.00; 1.23)	0.98 (0.86; 1.13)	1.23 (1.17; 1.31)	0.002
Lower limbs BMC (g)	893.00 (739.40; 1002.30)	692.00 (605.60; 811.46)	1036.00 (977.53; 1138.97)	<0.001
Spine BMD (g/cm2)	1.19 (1.13; 1.26)	1.16 (1.01; 1.26)	1.20 (1.12; 1.33)	0.127
Spine BMC (g)	206.00 (189.06; 229.94)	192.50 (150.87; 225.83)	228.00 (201.79; 272.62)	0.036
Trunk BMD (g/cm2)	0.95 (0.72; 0.99)	0.94 (0.80; 0.97)	0.99 (0.92; 1.01)	0.040
Trunk BMC (g)	724.00 (657.36; 781.81)	668.50 (494.48; 709.29)	791 (735.13; 828.76)	0.001
Ribs BMD (g/cm2)	0.75 (0.73; 0.75)	0.72 (0.64; 0.77)	0.78 (0.74; 0.84)	0.018
Ribs BMC (g)	202.00 (180.53; 240.17)	181.50 (164.08; 199.25)	244.00 (203.59; 272.17)	0.005
Pelvis BMD (g/cm2)	0.95 (0.89; 1.03)	0.89 (0.80; 1.04)	0.96 (0.92; 1.06)	0.138
Pelvis BMC (g)	286.00 (248.53; 306.70)	245.50 (195.58; 287.88)	309.00 (289.18; 341.88)	0.001
RSMI (Kg/m2)	6.44 (6.22; 7.20)	6.06 (4.88; 6.42)	7.16 (6.49; 7.45)	0.004
Total lean mass (g)	44428.00 (39596.91; 49777.45)	35209.00 (31745.77; 39766.96)	49942.00 (48203.12; 53897.11)	<0.001
Total fat mass (g)	23723.00 (20164.15; 27311.36)	27347.00 (22485.54; 33740.25)	20449.00 (13788.75; 24991.86)	0.037
Total bone mass (g)	2398.00 (2181.28; 2663.11)	2050.00 (1801.29; 2239.13)	2681.00 (2529.86; 2909.43)	<0.001

NET, neuroendocrine tumor; DXA, dual-energy X-ray absorptiometry scan; BMD, bone mineral density; BMC, bone mineral content; TBS, trabecular bone score; FN, femoral neck; RSMI, relative skeletal muscle index; n, number; CI, confidence interval.

### Correlation of DXA-derived parameters with NET aggressiveness

Additional within-group analyses were performed in the NET cohort after stratifying patients by disease stage at diagnosis, tumor grade, and treatment approach.

**Figure 1:**
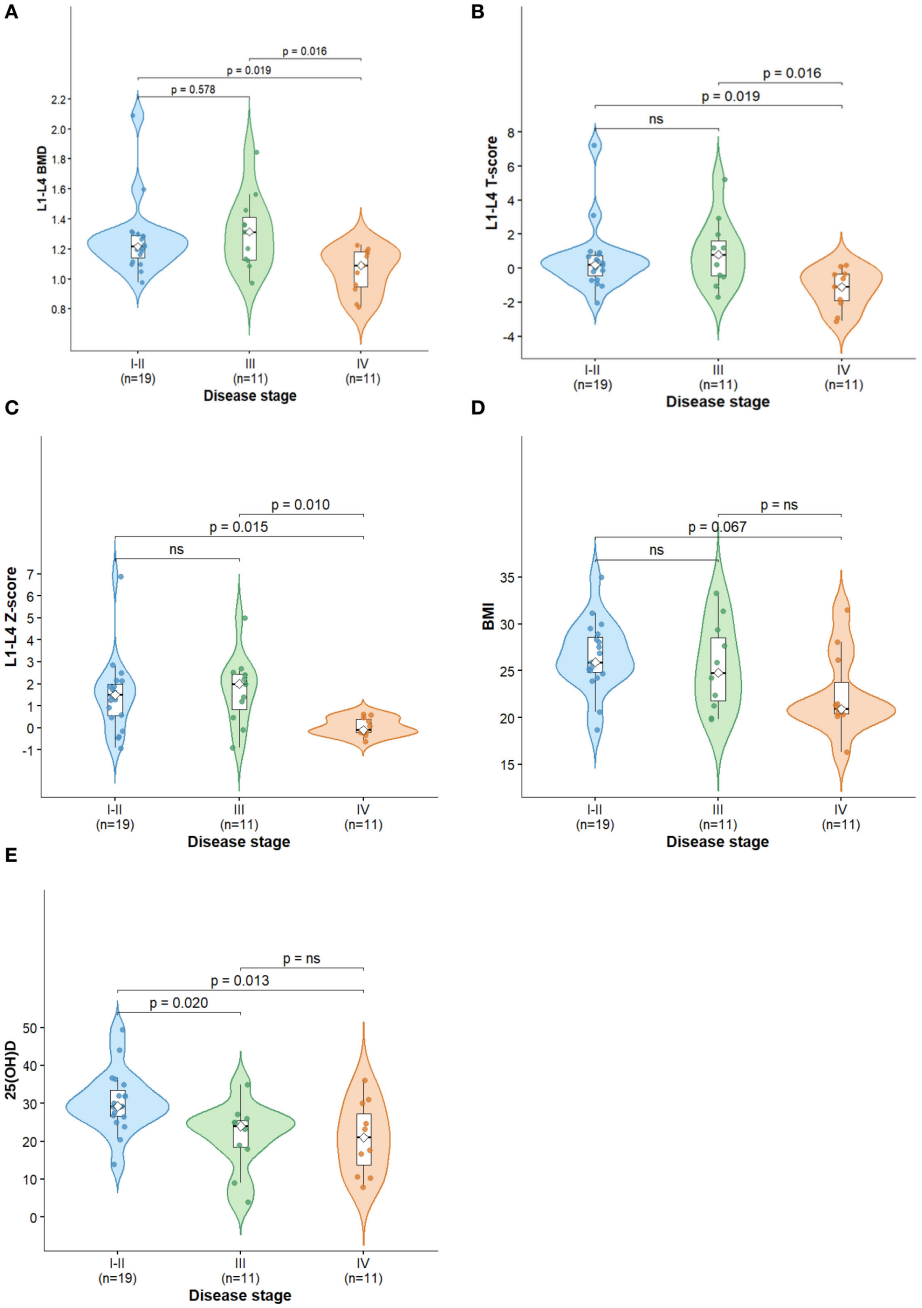
Violin plots showing the comparison according to disease stage of **(A)** L1-L4 BMD (bone mineral density); **(B)** L1-L4 T-score; **(C)** L1-L4 Z-score; **(D)** BMI (body mass index) and **(E)** 25(OH)D (25-hydroxyvitamin D) levels. Statistical comparisons are reported with the corresponding p-values. ns, not statistically significant.

Patients with stage IV NETs presented significantly lower values of L1-L4 BMD, L1-L4 T-score and L1-L4 Z-score compared with both stage III and stage I-II NETs. Moreover, stage IV and III NETs also exhibited inferior levels of 25(OH)D than stage I-II (p=0.013 and p=0.020, respectively). Lower BMI levels were observed in stage IV than stage I-II NETs (p=0.067) (**Figure 1**).

**Figure 2:**
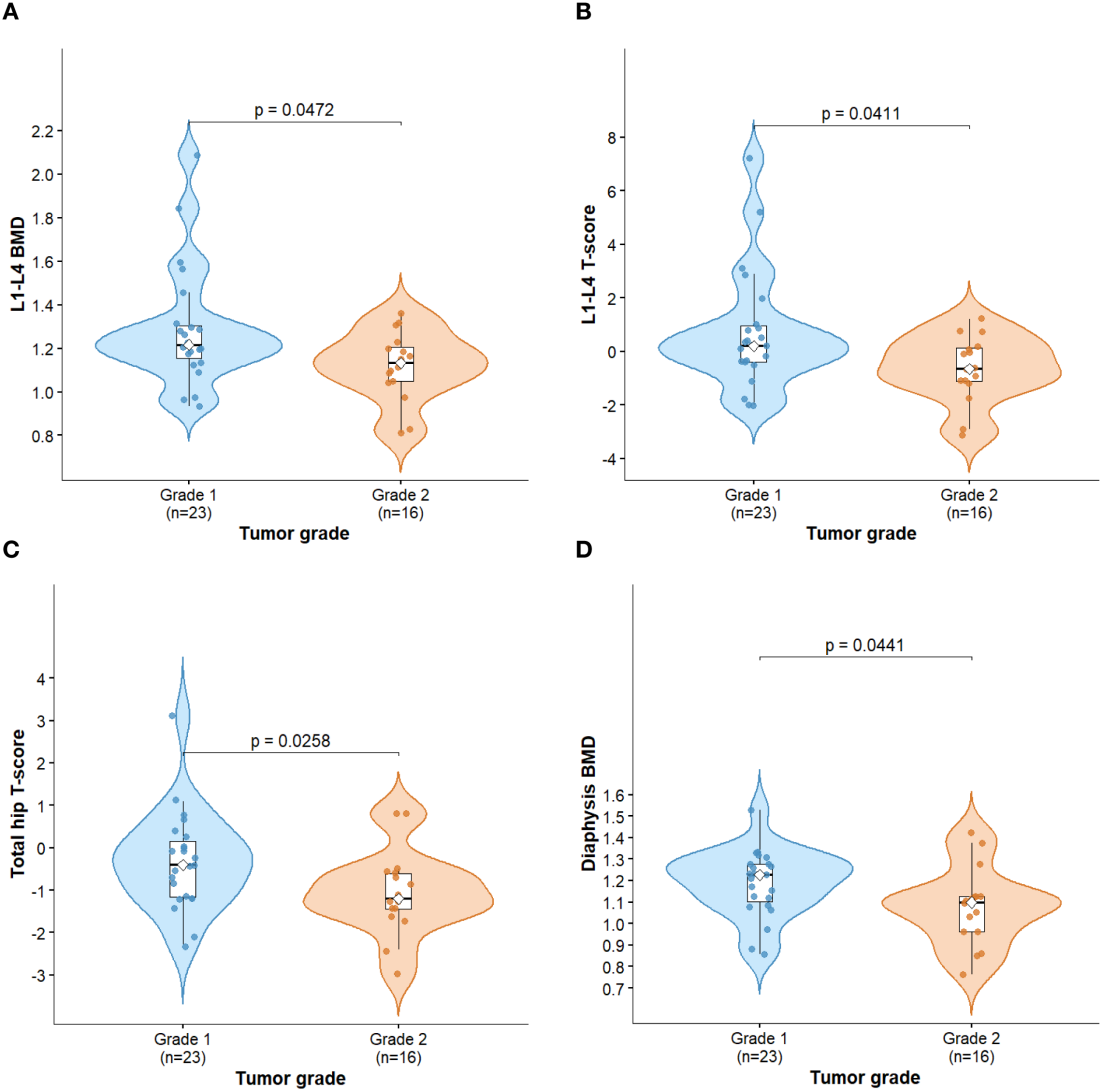
Violin plots showing the comparison according to tumor grade of **(A)** L1-L4 BMD (bone mineral density); **(B)** L1-L4 T-score; **(C)** total hip T-score and **(D)** diaphysis BMD. Statistical comparisons are reported with the corresponding p-values.

From tumor grade-based analysis, G2 NETs exhibited significantly lower L1-L4 BMD (p=0.047), L1-L4 T-score (p=0.041), total hip T-score (p=0.026) and diaphysis BMD (p=0.044) than G1 NETs. Moreover, a trend toward lower FN BMD (p=0.098), FN T-score (p=0.074), L1-L4 Z-score (p=0.092), total hip BMD (p=0.082), total hip Z-score (p=0.097) and trochanter T-score (p=0.086) was observed in G2 compared to G1 NETs (**Figure 2**).

**Figure 3:**
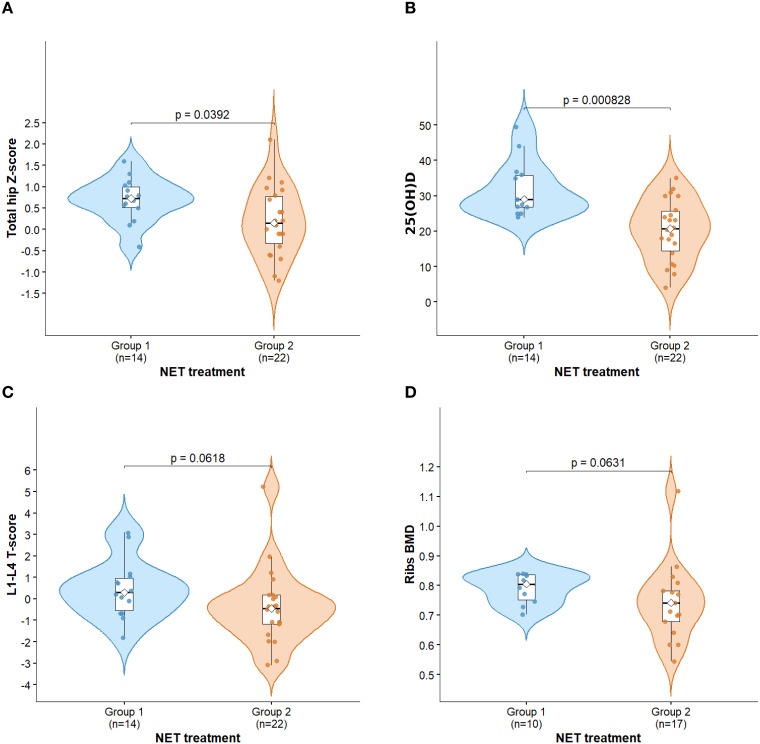
Violin plots showing the comparison between group 2 (somatostatin analogues/radioligand therapy after surgery) and group 1 (surgical treatment alone) regarding **(A)** total hip Z-score; **(B)** 25(OH)D (25-hydroxyvitamin D); **(C)** L1-L4 T-score and **(D)** ribs bone mineral density (BMD). Statistical comparisons are reported with the corresponding p-values.

As regards NET-related treatment, patients receiving surgery followed by SSA and/or RLT (group 2) presented significantly inferior total hip Z-score (p=0.039), 25(OH)D levels (p<0.001) and BMI (p=0.036) in comparison with their counterparts treated with surgery alone (group 1). Lower values of L1-L4 T-score (p=0.062) and ribs BMD (p=0.063) were also documented in group 2 compared to group 1, achieving almost statistical significance. No further statistically relevant differences were observed in relation to NET aggressiveness and therapy (**Figure 3**).

### Univariate and multivariate Cox regression analysis

At univariate Cox regression analysis, significant associations were observed between longer PFS and higher levels of 25(OH)D (HR 0.933, 95% CI: 0.880-0.989; p=0.019) and BMI (HR 0.815, 95% CI: 0.706-0.940; p=0.005). In contrast, lower PFS correlated with increased values of Ki-67 index (HR 1.064, 95% CI: 1.016-1.114; p=0.008), advanced stage at diagnosis (HR 4.575, 95% CI: 1.307-16.016; p=0.018), functioning NETs (HR 5.314, 95% CI: 1.475-29.150; p=0.011), requirement of medical therapy (SSA/RLT) after surgery (HR 4.412, 95% CI: 1.008-19.312; p=0.050), presence of bone fractures (HR 4.053, 95% CI: 1.170-14.038; p=0.028) and of bone metastasis (HR 5.262, 95% CI: 1.418-19.519; p=0.014), and L1-L4 osteoporosis (HR 6.691, 95% CI: 1.335-33.544; p=0.022). A trend of a positive correlation between better PFS and vitamin D supplementation (HR 0.394, 95% CI: 0.146-1.060; p=0.067) and higher trochanter T-score (HR 0.650, 95% CI: 0.394-1.070; p=0.092) was observed. In contrast, the presence of total hip osteoporosis was negatively associated with PFS, without achieving statistical significance (HR 7.496, 95% CI: 0.844-66.552; p=0.072).

In the multivariate analysis, higher levels of 25(OH)D (HR 0.844, 95% CI: 0.741-0.961; p=0.011) and BMI (HR 0.596, 95% CI: 0.359-0.990; p=0.047) were confirmed as independent predictors of longer PFS, while increased Ki-67 index was associated with shorter PFS (HR 1.178, 95% CI: 1.034-1.342; p=0.014) (**Table 4**).

**Table 4:** Univariate and multivariate Cox regression analysis.

Variable	Univariate analysis		Multivariate analysis	
	HR (95% CI)	p-value	HR (95% CI)	p-value
25(OH)D (continuous variable)	0.933 (0.880-0.989)	0.019	0.844 (0.741-0.961)	0.011
Vitamin D supplementation	0.394 (0.146-1.060)	0.067		
BMI (continuous variable)	0.815 (0.706-0.940)	0.005	0.596 (0.359-0.990)	0.047
Age at NET diagnosis	1.010 (0.959-1.062)	0.728		
Sex=male	1.407 (0.502-3.941)	0.518		
Primary tumor dimension	1.009 (0.979-1.040)	0.570		
Ki-67 index (continuous variable)	1.064 (1.016-1.114)	0.008	1.178 (1.034-1.342)	0.014
Stage at diagnosis III-IV	4.575 (1.307-16.016)	0.018		
Functioning NET	5.314 (1.475-29.150)	0.011		
NET therapy = surgery followed by SSA/RLT	4.412 (1.008-19.312)	0.050		
Presence of bone fractures	4.053 (1.170-14.038)	0.028		
Presence of BM at diagnosis and during follow-up	5.262 (1.418-19.519)	0.014		
HbA1c	0.954 (0.548-1.662)	0.868		
Glycemia	0.998 (0.985-1.011)	0.771		
TGL	0.993 (0.982-1.004)	0.219		
LDL cholesterol	0.999 (0.990-1.009)	0.892		
HDL cholesterol	1.005 (0.978-1.033)	0.710		
Total cholesterol	0.999 (0.990-1.008)	0.789		
Lumbar spine osteoporosis	6.691 (1.335-33.544)	0.022		
Lumbar spine osteopenia	1.613 (0.538-4.835)	0.396		
L1-L4 BMD	0.614 (0.064-5.888)	0.674		
L1-L4 BMC	0.998 (0.978-1.018)	0.838		
L1-L4 T-score	0.930 (0.696-1.242)	0.624		
L1-L4 Z-score	0.959 (0.692-1.330)	0.804		
FN BMD	1.008 (0.011-90.024)	0.997		
FN BMC	1.108 (0.722-1.698)	0.642		
FN T-score	0.918 (0.511-1.649)	0.775		
FN osteopenia	0.764 (0.250-2.338)	0.639		
FN osteoporosis	2.443 (0.629-9.492)	0.199		
FN Z-score	0.936 (0.516-1.698)	0.828		
Total hip BMD	0.178 (0.004-7.150)	0.362		
Total hip BMC	0.980 (0.916-1.049)	0.562		
Total hip T-score	0.869 (0.519-1.454)	0.595		
Total hip osteopenia	1.110 (0.394-3.127)	0.845		
Total hip osteoporosis	7.496 (0.844-66.552)	0.072		
Total hip Z-score	0.686 (0.364-1.293)	0.246		
Trochanter BMD	0.180 (0.006-5.560)	0.330		
Trochanter BMC	0.940 (0.819-1.079)	0.383		
Trochanter T-score	0.650 (0.394-1.070)	0.092		
Trochanter Z-score	0.804 (0.494-1.309)	0.383		
Diaphysis BMD	0.156 (0.007-3.448)	0.242		
Diaphysis BMC	0.963 (0.825-1.124)	0.633		
Head BMD	0.721 (0.095-5.465)	0.753		
Head BMC	1.003 (0.998-1.008)	0.221		
Upper limbs BMD	1.109 (0.018-69.657)	0.961		
Upper limbs BMC	1.002 (0.995-1.008)	0.610		
Lower limbs BMD	1.337 (0.059-30.317)	0.856		
Lower limbs BMC	1.001 (0.998-1.004)	0.494		
Trunk BMD	0.204 (0.002-23.554)	0.514		
Trunk BMC	0.999 (0.996-1.003)	0.706		
Ribs BMD	0.285 (0.002-50.324)	0.636		
Ribs BMC	0.997 (0.988-1.007)	0.601		
Pelvis BMD	0.229 (0.002-21.389)	0.526		
Pelvis BMC	1.002 (0.992-1.011)	0.725		
Spine BMD	0.280 (0.012-6.550)	0.431		
Spine BMC	0.996 (0.986-1.005)	0.384		
RSMI	0.758 (0.477-1.207)	0.246		
Total bone mass	1.000 (0.999-1.002)	0.604		
Total fat mass	1.000 (1.000-1.000)	0.046		
Total lean mass	1.000 (0.9999-1.0001)	0.857		

NET, neuroendocrine tumor; 25(OH)D, 25-hydroxyvitamin D; BMI, body mass index; SSA, somatostatin analogue; RLT, radioligand therapy; BM, bone metastasis; HbA1c, glycated hemoglobin; TGL, triglycerides; HDL, high-density lipoprotein; LDL, low-density lipoprotein; BMD, bone mineral density; BMC, bone mineral content; FN, femoral neck; RSMI, relative skeletal muscle index; HR, hazard ratio; CI, confidence interval.

### Kaplan-Meier survival analysis for PFS

**Figure 4:**
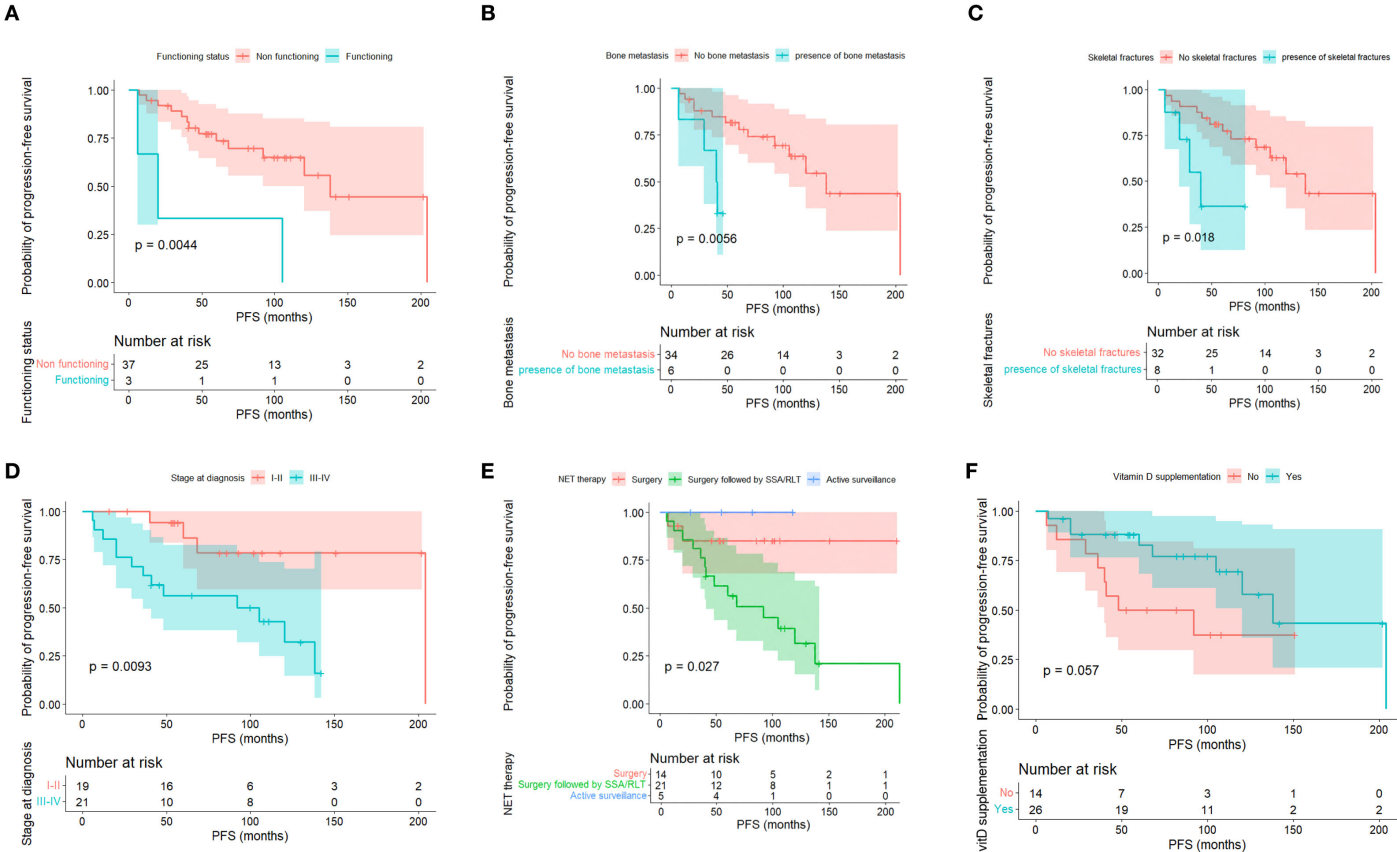
Kaplan–Meier curves for progression-free survival (PFS) according to **(A)** NET (neuroendocrine tumor) functioning status, **(B)** bone metastasis, **(C)** skeletal fractures, **(D)** NET stage at diagnosis (I-II vs III-IV), **(E)** NET therapy, **(F)** vitamin D supplementation. Statistical comparisons are reported as log-rank p-values.

Kaplan-Meier analysis showed a significant difference in PFS according to functioning status of NETs (log-rank p=0.004), presence of bone metastasis (log-rank p=0.006) and skeletal fractures (log-rank p=0.018), disease stage at diagnosis (log-rank p=0.009) and NET therapy (log-rank p=0.027) (**Figures 4A–E**).

Although not achieving statistical significance, shorter PFS was observed in patients with G2 versus G1 NET (log-rank p=0.25), lumbar osteopenia or osteoporosis (log-rank p=0.14), 25(OH)D levels < 20 ng/mL (log-rank p=0.1), and absent vitamin D supplementation (p=0.057) **Figure 4F**).

Given the limited sample size and low number of events, Kaplan-Meier analyses for other categorized variables such as BMI and L1–L4 TBS were considered non-informative, as stratification led to small subgroup sizes and unstable estimates. No significant differences in PFS were observed for the glycolipidic profile, namely glycated hemoglobin and triglycerides, when categorized.

### Correlations between Ki-67 index, clinical variables and DXA-derived parameters

Spearman’s correlation was applied to Ki-67 index, age at NET diagnosis, age at study enrolment, and HbA1c, as these variables were not normally distributed according to the Shapiro–Wilk test. For BMI, either Spearman’s (rho) or Pearson’s (r) correlation was used depending on the distribution of the other continuous variables.

Ki-67 index was negatively associated with total hip T-score (rho= −0.314, p=0.048) and diaphysis BMD (rho= -0.354, p=0.025); whereas a modest but direct correlation between Ki-67 index and primary NET dimension (rho=0.311, p=0.051) was found. No additional statistically significant correlations were found between Ki-67 index and the other DXA-derived parameters, 25(OH)D levels, or BMI.

Patient’s age at NET diagnosis negatively correlated with FN T-score (rho= -0.355, p=0.023), FN BMD (rho= -0.396, p=0.010), FN BMC (rho= -0.409, p=0.008), total hip BMD (rho= -0.455, p=0.003), total hip BMC (rho= -0.454, p=0.003), trochanter BMD (rho= -0.357, p=0.022), trochanter BMC (rho= -0.369, p=0.018), trochanter T-score (rho = -0.328, p= 0.036), diaphysis BMD (rho= -0.499, p=0.001), diaphysis BMC (rho= -0.519, p=0.001), and pelvis BMD (rho= -0.366, p=0.051).

Patient’s age at study enrolment negatively correlated with diaphysis BMD (rho= -0.332, p=0.034), diaphysis BMC (rho= -0.299, p=0.057), pelvis BMD (rho= -0.476, p=0.009). No further statistically significant associations were observed.

BMI positively correlated with L1-L4 BMD (rho=0.580, p<0.001), L1-L4 BMC (rho=0.382, p=0.014), L1-L4 T-score (rho=0.583, p<0.001), L1-L4 Z-score (rho=0.413, p=0.007), trunk BMD (r=0.629, p<0.001), trunk BMC (r=0.521, p=0.004), spine BMD (r=0.706, p<0.001), spine BMC (r=0.611, p<0.001), ribs BMD (rho=0.551, p=0.002), ribs BMC (r=0.472, p=0.010), pelvis BMD (r=0.431, p=0.020), RSMI (rho=0.416, p=0.025), total fat mass (r=0.846, p<0.001), 25(OH)D (r=0.320, p=0.042); BMI inversely correlated with primary NET dimension (rho= -0.345, p=0.027).

No significant correlations were found between HbA1c and DXA parameters, Ki-67 or vitamin D levels.

### Comparison of DXA parameters between NET patients and CTs

The control group comprised 47 subjects, including 25 women (53%) and 22 men (47%), with a mean age of 69.3 ± 11.8 years and a mean BMI of 25.9 ± 4.4 Kg/m². Among the 41 NET patients, a subgroup of 38 individuals comparable to the control group for sex, age, and BMI was selected for the matched-control comparison (**Table 5**).

**Table 5:** Comparison between NET patients and CTs regarding demographic and anthropometric data.

Variable		NET patients (n=38)	CTs (n=47)	P-value
Sex N (%)	Female	19 (50%)	25 (53%)	0.6
	Male	19 (50%)	22 (47%)	
Age, mean ± SD (yo)		70.3 ± 8.4	69.3 ± 11.8	0.3
BMI, mean ± SD (Kg/m^2^)		25.2 ± 4.4	25.9 ± 4.4	0.7

NET, neuroendocrine tumor; CTs, control group subjects; BMI, body mass index; SD, standard deviation; n, number; yo, years old.

**Figure 5:**
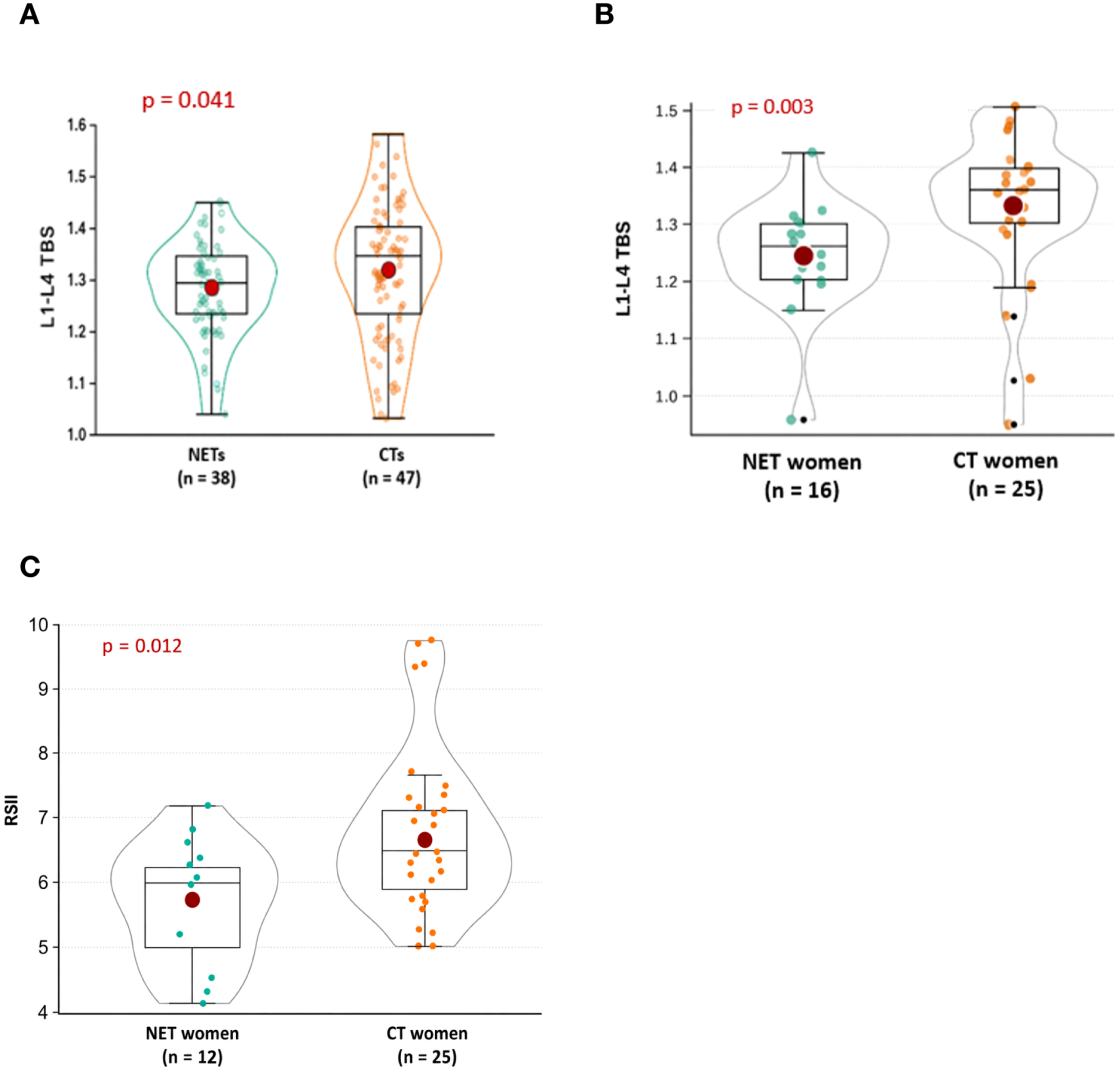
Violin plots showing the comparison of **(A)** L1-L4 TBS (trabecular bone score) between NET (neuroendocrine tumor) patients and CTs (control group subjects), **(B)** L1-L4 TBS between NET women and CT (control group) women, **(C)** RSMI (relative skeletal muscle index) between NET women and CT women. Statistical comparisons are reported with the corresponding p-values.

NET patients presented significantly lower L1-L4 TBS than CTs (median 1.29 vs 1.36, p=0.041). No significant differences were observed between the 2 groups in terms of BMD, T-score or Z-score at the lumbar and femoral sites. In a sex-stratified analysis, in the female subgroups, NET patients exhibited significantly inferior L1-L4 TBS values (median 1.26 vs 1.36, p=0.003) and RSMI (median 6.06 vs 6.53, p=0.012) when compared to CTs. In contrast, among males no statistically relevant differences were observed between NET individuals and CTs in either DXA-derived bone or body composition parameters (**Figure 5**).

## Discussion

This study explored the association between NET-related disease burden and musculoskeletal health, as well as the potential prognostic relevance of bone impairment and vitamin D levels in patients with GEP and lung NETs. Given the limited evidence in this field, standardized recommendations for the assessment and management of bone and muscle health in NET populations are yet to be established.

In the present cohort, NET patients showed a high prevalence of skeletal impairment, characterized by degraded L1–L4 TBS in 71% of cases and reduced bone mass, mainly osteopenia, affecting up to 59% of patients at the femoral neck. Although skeletal fractures were recorded in 20% of patients, their retrospective collection did not allow reliable classification according to fragility-related nature, trauma mechanism, anatomical site, timing or radiological confirmation. Therefore, fracture-related findings should be interpreted cautiously. Bone metastases were observed in 15% of cases, and only one patient had BM at NET diagnosis. These results suggest that skeletal impairment in NET patients may frequently occur independently of overt metastatic bone involvement. Overall, in the neuroendocrine setting, BM have been reported in less than 15% of cases (8, 9, 14, 15); however, our findings showed lower rates when compared to the Italian multicenter retrospective study, including 320 NEN patients (151 GEP, 117 lung, 52 of other origin) with bone being the first metastatic site in 41% of cases (9).

Compared with age-, sex-, and BMI-matched controls, NET subjects showed significantly lower TBS despite comparable BMD, T-score, and Z-score values. Interestingly, in the female subgroup, NET patients had lower TBS and RSMI than matched controls, suggesting a potential sex-specific vulnerability that requires confirmation in larger cohorts. The requirement of serum 25(OH)D levels >30 ng/mL in controls was intentionally chosen to ensure a healthy, vitamin D-replete reference group and to better capture potential alterations in vitamin D status associated with NET. We acknowledge that this criterion may have contributed to the observed TBS difference between NET patients and CTs, and prevented vitamin D-adjusted sensitivity analyses, as sufficiency was part of the control-group definition. However, vitamin D status was deliberately left unrestricted in NET individuals, since hypovitaminosis D may reflect the nutritional and systemic burden of the oncological disease. Therefore, preserving the full spectrum of vitamin D levels in the NET cohort allowed us to explore a real-world association between vitamin D status, bone health, NET aggressiveness and progression-free survival.

Furthermore, within the NET group, advanced disease stage, higher grade (G2 vs G1) and systemic oncologic therapies (SSA/RLT) were associated with poorer bone health. In survival analyses, several clinical and skeletal variables, including bone metastases, skeletal fractures, L1–L4 osteoporosis, higher Ki-67 index, advanced stage, functioning NETs, and the need for SSA/RLT, were associated with shorter PFS at univariate analysis. However, after multivariable adjustment, only Ki-67 remained independently associated with poorer PFS, whereas higher 25(OH)D levels and BMI were independently associated with longer PFS. Both 25(OH)D and BMI were significantly reduced in patients with advanced-stage disease and in those receiving SSA/RLT after surgery. Overall, these findings suggest that vitamin D status and nutritional and body composition parameters may have prognostic relevance in NET patients, although their causal role cannot be inferred from the present retrospective observational design. As BMI and 25(OH)D levels were collected within 6 months of DXA assessment and not necessarily at NET diagnosis, these variables should be interpreted as outcome-associated parameters rather than baseline predictors of PFS. Furthermore, reverse causality cannot be excluded, as lower BMI and vitamin D may have resulted from more advanced disease, malabsorption, nutritional deterioration, or systemic treatment exposure.

As regards the association between higher BMI and prolonged PFS, several underlying mechanisms may be involved, including greater nutritional reserve, lower burden of cancer-related catabolism, and/or an obesity-paradox phenomenon. However, BMI does not accurately reflect body composition, as it does not discriminate between lean and fat mass, nor does it capture the distribution of adipose tissue across subcutaneous and visceral compartments. Therefore, given these limitations, BMI could be better regarded as a marker of nutritional-metabolic status rather than as a precise measure of body composition. In light of these considerations, future studies incorporating more refined methods, including bioelectrical impedance analysis and imaging-based body composition assessment (i.e. computed tomography), are warranted to clarify the potential prognostic role of body composition with specific regard to adipose and muscle tissue compartments in NET patients.

The modest inverse correlations observed between Ki-67 index and total hip T-score and diaphyseal BMD, may suggest an association between higher tumor proliferative activity and poorer cortical/appendicular bone status. However, no correlations were found across other DXA-derived parameters, L1-L4 TBS, or vitamin D levels, thus indicating that these findings should be interpreted cautiously and regarded as exploratory. Age at NET diagnosis showed more consistent inverse correlations with several bone parameters, supporting the role of the patient’s age as a relevant determinant of skeletal impairment in this population and as a potential confounding factor. Conversely, BMI was positively associated with several BMD and BMC parameters, as well as RSMI and 25(OH)D levels, suggesting that better nutritional and body composition status may contribute to preserving both bone and muscle health in patients with NETs.

Consistent with our findings, emerging evidence suggests that impaired bone health may be frequent in NET patients, irrespective of skeletal metastatic involvement. Aktypis et al. reported a 3-fold higher risk of osteopenia or osteoporosis in GEP-NET patients, predominantly with localized disease, compared with sex- and age-matched controls, with gastric NETs showing the lowest bone mass (16). In a retrospective study including 91 panNET patients, osteoporosis and low bone mass, assessed by computed tomography attenuation, were documented in 37.4% and 60.4% of cases, respectively, with osteoporosis reaching 64.0% among patients older than 50 years. Moreover, age and diabetes were identified as risk factors for bone loss, while no correlation was found between tumor grade and osteoporosis (17). Among GEP-NETs, Benevento et al., further showed that patients with gastric NETs had a higher prevalence of osteopenia/osteoporosis (respectively 61.5%, 8/13 vs 15.4%, 2/13; p=0.04) and vitamin D deficiency (92.3% vs 46.2%, p = 0.03) than those with entero-pancreatic NETs, thereby requiring more intensive cholecalciferol supplementation (18). In patients with NETs (n=46) and carcinoid syndrome, Gupta et al. reported osteopenia and osteoporosis in 32.6% and 41.3% of cases, respectively, and found a significant correlation between higher urinary 5-hydroxyindoleacetic acid (5-HIAA) levels and lower hip BMD; however, urinary 5-HIAA was not confirmed as an independent predictor of BMD (19). Evidence in carcinoid syndrome remains inconsistent, as in other studies no significant differences in bone metabolism were reported between patients with carcinoid syndrome and controls (20, 21). More recently, Brunetti et al. highlighted the clinical relevance of skeletal fragility already at NET diagnosis in a retrospective cohort of 291 patients with G1–G2 GEP-NETs. Bone metastases were present in 17% of patients with distant metastases at diagnosis, fragility fractures were identified in 15.5% of patients at baseline, and new fractures occurred in an additional 10.5% during follow-up, particularly among those with prior fractures and oncologic surgical treatment. After adjustment for major confounders, GEP-NET patients showed a higher fracture risk than age-matched controls, while older age and severe 25(OH)D deficiency (<10 ng/mL) emerged as independent predictors of fracture risk (22).

As regards bone quality assessed by TBS, in the present study, a substantial prevalence of trabecular bone deterioration was observed regardless of BMD; however, no significant correlations were found between lower TBS and greater neoplastic aggressiveness expressed in terms of histological grade, advanced disease stage or requirement of systemic medical treatment. TBS is a DXA-derived additional index of skeletal fragility, which may detect earlier deterioration of trabecular microarchitecture not adequately captured yet by BMD alone. Its added value has been documented in several forms of secondary osteoporosis, characterized by a potential discrepancy between BMD and fracture risk (23). In oncology, TBS decline has been mainly described in breast cancer patients receiving aromatase inhibitors (AI), prostate cancer patients undergoing androgen deprivation therapy, and patients with differentiated thyroid cancer (DTC) exposed to long-term TSH (Thyroid-Stimulating Hormone) suppression (24–27). Notably, in the setting of breast cancer treated with AI (24) and DTC (27), TBS deterioration has been observed independently of BMD, and has improved fracture risk stratification when combined with BMD and FRAX (Fracture Risk Assessment Tool) (24). Within the neuroendocrine field, however, evidence specifically addressing TBS remains scarce and is largely limited to pheochromocytoma and paraganglioma (PPGL). In PPGL patients, lower TBS has been reported together with reduced BMD and increased bone resorption markers (C-terminal telopeptide), with partial improvement after tumor resection (28, 29). Notably, TBS was inferior in patients with vertebral fractures (VF) regardless of lumbar spine BMD, suggesting a major role of impaired bone quality rather than bone mass in the occurrence of VF in this context (30).

Regarding vitamin D, a relevant prevalence of inadequate levels has been repeatedly documented in NET patients (18, 22, 31–36). In this analysis, hypovitaminosis D (<30 ng/mL) was found in 68% of cases, with insufficient and deficient levels recorded in 44% and 24% of patients, respectively. Lower 25(OH)D concentration significantly correlated with major NET aggressiveness, namely advanced disease stage and need for medical therapy (SSA/RLT), but not with Ki-67 index. This discrepancy with our previous larger retrospective study (31), in which vitamin D levels <20 ng/mL were associated with higher Ki-67, may be attributable to the smaller sample size and limited heterogeneity in Ki-67 values (median 2%; 95% CI 1-3) in the present cohort. Notably, higher vitamin D levels were associated with better PFS at univariate analysis and confirmed as an independent predictor of longer PFS alongside BMI in this study, while vitamin D supplementation showed a univariate trend of association toward improved PFS (p=0.067). These findings are consistent with previous retrospective studies suggesting an association between vitamin D status, tumor aggressiveness and prognosis in NET patients. Lower vitamin D levels have been associated with disease progression and higher tumor grade and shorter PFS, although their impact on the survival outcomes has not been consistently demonstrated (31, 32, 34). Moreover, vitamin D supplementation has also been associated with improved OS at least in one GEP-NET retrospective cohort (34).

Overall, most of the available evidence in the neuroendocrine setting, which remains limited and entirely retrospective, appears suggestive of a negative impact of hypovitaminosis D on NET prognosis; however, low vitamin D levels may reflect not only a biological contributor to tumor aggressiveness, but also a surrogate marker of malnutrition, impaired gastrointestinal absorption or greater disease burden. In the present study, we could not determine whether lower 25(OH)D levels were primarily caused by reduced intake, malabsorption, pancreatic exocrine insufficiency, previous gastrointestinal surgery, or greater disease burden, as detailed information regarding nutritional markers (g.e. albumin, prealbumin, micronutrients) or pancreatic insufficiency was not systematically available for all patients. Therefore, additional and more robust studies, including also dedicated data on these variables, are warranted to better characterize hypovitaminosis D and to clarify its potential prognostic relevance in neuroendocrine neoplasms. Evidence across other malignancies has been supportive of correlations of hypovitaminosis D with oncogenesis, progression and survival outcomes (37, 38). Such data were supported by the numerous antitumorigenic properties of vitamin D documented in preclinical research, ranging from the modulation of cancer cell proliferation and differentiation, apoptosis and autophagy, to angio genesis, migration and invasion of cancer cells (39, 40).

The underlying pathophysiological mechanisms of bone loss in patients with NETs, beyond the development of bone metastases, are not fully elucidated. However, the vulnerability of NET patients to skeletal fragility may reflect multifactorial interplays including tumor-related hormonal syndromes, oncological treatments, nutritional impairment, vitamin D deficiency, inflammation and specific microRNAs (miRNAs) (10). In functioning NETs, biologically active mediators such as serotonin, and more rarely ectopic adrenocorticotropic hormone (ACTH) and parathyroid hormone-related peptide (PTHrP) secretion, may disrupt bone homeostasis. Chronic gastrointestinal symptoms (nausea, vomiting, diarrhea, steatorrhea, malabsorption) related to hormone hypersecretion (serotonin, VIP, calcitonin, gastrin) or therapeutic approaches (surgery, SSA, target therapies, chemotherapy) may compromise patients’ nutritional status, calcium and vitamin D intake and absorption, thus promoting bone deterioration. A chronic pro-inflammatory environment and several miRNAs (miRNA-210, miRNA-21, miRNA-196a), which have been implicated both in NET development and progression and bone remodeling, may be additional plausible contributors to NET-related skeletal fragility; however, given the lack of dedicated studies, such interplay remains speculative (10, 41–45). In case of NETs arising within MEN1 syndrome, the concurrence of PHPT provides another relevant mechanism of early bone impairment in this population (10). In our study, we excluded patients with NETs arising within hereditary syndromes, given the higher prevalence of PHPT with subsequent impact on bone homeostasis.

Overall, considering the generally prolonged survival of NET patients, the cumulative exposure to the above-mentioned factors may lead over time to a clinically meaningful risk of bone impairment.

Specifically regarding SSAs, preclinical evidence suggests a possible direct interference with bone metabolism, besides their indirect effects attributable to pancreatic exocrine insufficiency and fat-soluble vitamin deficiency (46–49). Previous reports have documented inhibitory properties of somatostatin on osteoprogenitor cell proliferation and differentiation, as well as on chondroprogenitor cell proliferation (48), and suppressive effects of octreotide (OCT) on the forskolin-stimulated adenylate cyclase activity in bone cell suspensions (49). More recently, in the study of Vitali et al. OCT was shown to exert SSTR2/5 (somatostatin receptor 2/5)-mediated anti-proliferative and pro-apoptotic actions on osteoblastic (OB) cells, and to inhibit the pancreatic NET cell migration toward OB-conditioned medium; thereby suggesting that the direct skeletal effects of OCT may preferentially involve osteoblast turnover and modulation of the tumor–bone microenvironment (46). Overall, the available data may support the biological plausibility of a direct SSA–bone interaction; however, the evidence remains sparse and still lacks validation in dedicated human studies.

In light of these considerations, the deterioration of the skeletal parameters observed in patients with advanced disease and in those receiving systemic therapy, i.e. SSAs and RLT, may be at least partly attributable to the multifactorial mechanisms described above. However, given the limited number of patients treated with RLT due to further disease progression, no reliable RLT-specific skeletal pattern could be identified. Therefore, the relationship between poorer bone health and systemic therapy should still be interpreted cautiously, as it may primarily reflect a more advanced disease stage rather than a direct treatment-related skeletal effect. Larger prospective studies, incorporating also the assessment of bone metabolism markers, are needed to clarify the potential contribution of neuroendocrine neoplasms and their treatments to bone health.

Regarding muscle evaluation, low DXA-derived RSMI values suggestive of sarcopenia were observed in 37% of NET patients. It is important to clarify that since muscle strength and physical performance were not assessed, a definitive diagnosis of sarcopenia could not be established according to EWGSOP criteria. In the present study, however, no statistically relevant correlations were documented between RSMI and tumor aggressiveness. Although no significant difference emerged between the overall NET cohort and controls, female NET patients showed lower RSMI values than matched controls. This finding may suggest a sex-specific vulnerability, whereby women, generally characterized by lower baseline lean mass and increased susceptibility to menopause-related muscle loss, may be more sensitive to the metabolic and nutritional burden of NETs. Accordingly, in the present NET cohort, female patients showed significantly lower RSMI and lean mass values than male patients. However, this observation remains speculative, given the small sample size, the lack of functional measures of muscle strength or performance, and the paucity of evidence in the neuroendocrine field, which preclude firm conclusions.

The considerable prevalence of low DXA-derived RSMI found in our cohort is generally plausible in cancer patients, including NETs, given the potential contribution of chronic gastrointestinal symptoms, malabsorption, and nutritional impairment, which may be experienced especially in advanced and aggressive oncological settings. In the study of Aktypis et al., inferior values of RSMI assessed by DXA, even though not suggestive of sarcopenia, were reported in GEP-NET patients in comparison with the control group (p<0.001). Moreover, within the NET group, RSMI significantly and positively correlated with lumbar spine and total hip BMD, and patients with low muscle mass also exhibited inferior total fat mass and fat mass ratio (16). In other studies exploring body composition via computed tomography, the prevalence of sarcopenia has been reported between 42.8% and 87.2% in GEP-NEN patients (50–55). Conversely, according to the standardized EWGSOP criteria used in the NUTRIGETNE study involving 399 patients with advanced GEP-NENs, a lower frequency was observed (15%) (36). At present, the prognostic impact of sarcopenia in the neuroendocrine setting remains inconclusive, due to the limited and retrospective nature of the available data, methodological biases, and heterogeneous findings. However, to date, the majority of evidence, mostly limited to GEP-NEN cohorts, appears supportive of a negative impact of sarcopenia or reduced skeletal muscle index on survival outcomes (PFS and/or OS) (50, 52, 56, 57).

Our study has several limitations to be acknowledged, including the small sample size and its retrospective design. The limited cohort size also prevented separate analyses according to primary tumor site between GEP and lung NETs. Furthermore, the number of patients across the examined subgroups and the relatively small number of progression events may have reduced the statistical power of subgroup comparisons and multivariable analyses; therefore, our findings should be regarded as exploratory and hypothesis-generating. In addition, the retrospective collection of data did not allow a reliable classification of fractures and systematic availability of bone turnover markers, thus limiting complementary insights into the skeletal impairment observed in this cohort. Finally, muscle assessment using DXA-derived RSMI alone does not adequately reflect muscle strength, quality or functional performance. Therefore, the incorporation of other evaluations, such as handgrip strength or imaging-based techniques, i.e. computed tomography for body composition analysis, is required to better characterize musculoskeletal alterations in NET patients.

Despite these limitations, this study has several strengths, including the matched-control design, the exclusion of major secondary causes of osteoporosis, and the integrated assessment of trabecular bone quality, bone mass, vitamin D status, muscle mass and survival outcomes. Importantly, our analysis specifically addressed skeletal health as an independent clinical issue in NET patients, beyond the presence of bone metastases. These preliminary findings highlighted the considerable prevalence of musculoskeletal impairment in NET patients, as well as the impact of NET-related disease burden on the bone mineralization parameters, and they support the need for specific recommendations to guide the assessment and management of muscle and bone health in clinical practice.

In conclusion, this study showed a relevant prevalence of musculoskeletal impairment in patients with GEP and lung NETs, characterized mainly by trabecular microarchitecture deterioration and femoral osteopenia, with TBS impairment detectable despite comparable BMD versus matched controls. Advanced disease stage, higher tumor grade and systemic treatment exposure (SSAs/RLT) were associated with poorer skeletal parameters. Despite the substantial prevalence of DXA-derived low muscle mass, no correlations were found between RSMI and tumor aggressiveness. Higher 25(OH)D levels and BMI were independently associated with longer PFS in this exploratory cohort, supporting a possible link between nutritional-metabolic status and oncological outcomes. Overall, although these findings remain hypothesis-generating, they support the clinical relevance of structured musculoskeletal assessment in NET patients. Larger prospective studies integrating the quantitative and qualitative evaluation of bone and muscle health, bone turnover markers, nutritional assessment, and survival outcomes are warranted to define evidence-based strategies for screening, prevention, and management of bone and muscle impairment in this population.

## Data Availability

The raw data supporting the conclusions of this article will be made available by the authors, without undue reservation.
